# Attitudes Toward Sex-Specific Versus Universal Newborn Screening for X-Linked Adrenoleukodystrophy in Hong Kong

**DOI:** 10.3390/ijns12030063

**Published:** 2026-07-31

**Authors:** Chloe Miu Mak, Hoi Ying Wu, Ariel Ying Wong, Felicite Enyu Song, Toby Chun Hei Chan, Cheuk Wing Fung, Suet Na Wong, Edgar Wai Lok Hau, Matthew Chun Wing Yeung

**Affiliations:** 1 Newborn Screening Laboratory, Department of Pathology, Hong Kong Children’s Hospital, Hong Kong SAR, China; 2 Metabolic Medicine Unit, Department of Pediatrics and Adolescent Medicine, Hong Kong Children’s Hospital, Hong Kong SAR, China; 3 Neurology Unit, Department of Pediatrics and Adolescent Medicine, Hong Kong Children’s Hospital, Hong Kong SAR, China; wongsn2@ha.org.hk; 4 Department of Clinical Genetics, Hong Kong Children’s Hospital, Hong Kong SAR, China

**Keywords:** X-linked adrenoleukodystrophy, peroxisomal disorder, gene therapy, parental attitude survey, newborn screening, *ABCD1* gene

## Abstract

X-linked adrenoleukodystrophy (X-ALD) is the most common peroxisomal disorder and is associated with serious clinical consequences. While newborn screening (NBS) could facilitate early identification and timely intervention, its implementation for X-ALD remains controversial due to ethical concerns arising from its X-linked recessive inheritance pattern, particularly regarding female newborns. This study aims to explore the perspectives of healthcare professionals and the general public on NBS for X-ALD in Hong Kong. An online survey with 20 quantitative questions on ethical considerations was conducted from May to August 2024. Among a total of 259 responses, most respondents (99.2%) supported NBS for their male newborns, primarily because it facilitates early diagnosis and effective management. The majority of the respondents were in favor of offering NBS to female newborns, citing potential benefits for the management of adult-onset disease, enhanced family planning and support, and opportunities for extended family screening. However, some respondents expressed concerns regarding (1) psychological stress and anxiety from uncertain disease onset and frequent monitoring; (2) potential genetic discrimination and adverse impact on insurance premiums/coverage; (3) affordability and accessibility of expensive treatments, such as gene therapy; and (4) ethical issues regarding children’s “right to an open future”, particularly for late-onset female X-ALD. To address these concerns while respecting family autonomy, we propose an opt-in system with clear, balanced information for parents, combined with a three-tier screening algorithm. In summary, while inclusion of X-ALD in Hong Kong’s NBS program receives strong community support, targeted measures are needed to mitigate the identified ethical and practical barriers.

## 1. Introduction

X-linked adrenoleukodystrophy (X-ALD) is the most common peroxisomal disorder worldwide. Reported birth incidence varies: 1 in 10,500 births in the USA [1], 1 in 17,000 in France [2], 1 in 9,900 in Italy [3], 1 in 6,236 in China [4], and 1 in 15,000 in Taiwan [5]. While affected male hemizygotes present early in childhood with cerebral adrenoleukodystrophy and/or primary adrenocortical insufficiency, female heterozygotes are generally asymptomatic throughout childhood and early adulthood. Female carriers typically begin to develop mild-to-moderate adrenomyeloneuropathy (AMN) in their 40s to 60s, but by the age of 60 years old, about 88% are affected [6,7].

The first newborn screening (NBS) program for X-ALD started in New York State, USA, in 2013, and X-ALD has been listed as a core condition in the Recommended Uniform Screening Panel (RUSP) since 2016. Nonetheless, uptake has been relatively slow, with NBS for X-ALD implemented in only 38 US states, as well as in the Netherlands, Taiwan, and Italy over the past decade [5,8,9,10]. Although the benefits for male newborns are well established, there have been ongoing ethical debates, primarily due to concerns that the condition in females is typically adult-onset and that effective treatments are lacking.

In Hong Kong, the public NBS encompasses inborn errors of metabolism diseases, classic galactosemia, congenital adrenal hyperplasia, severe combined immunodeficiency and spinal muscular atrophy, glucose-6-phosphate dehydrogenase deficiency, and congenital hypothyroidism. X-ALD, however, is not yet included within the existing screening scope. This study aims to explore medical and non-medical stakeholders’ views and attitudes towards NBS for X-ALD, thereby delineating key concerns regarding its potential inclusion and providing foundational evidence to inform future policy development on expanding the NBS program.

## 2. Materials and Methods

A prospective cross-sectional online survey was conducted targeting the local general population and healthcare professionals, aged 18 or above, between 1 May 2024 and 31 August 2024. The survey was conducted using a free-to-use survey maker website (qualtrics.com, Qualtrics, LLC, Provo, UT, USA); anonymized responses were collected after informed consent was obtained. The questionnaire was distributed via email and shared through social media platforms and communication applications to both the general population and healthcare professionals via the researchers’ connection. 

The questionnaire was a modified version of a published survey of NBS for X-ALD conducted in the Netherlands [11]. The questionnaire’s design has been refined and also translated into a traditional Chinese version by a multi-disciplinary team of experts in the field of NBS and clinical management for X-ALD.

The questionnaire consisted of twenty questions (Supplementary File S1):

- Questions 1–5: Background knowledge of the respondent towards X-ALD.
- Questions 6–7: Respondents’ views on NBS of X-ALD for male newborns.
- Questions 8–10: Respondents’ views on NBS of X-ALD for female newborns.
- Question 11: Respondents’ preferences on whether to offer their parents the right to give consent for NBS for X-ALD with different disease onsets.
- Question 12: Respondents’ opinions on the impact of regular testing and monitoring for screened-positive patients with different disease onsets.
- Question 13: Respondents’ opinions on reasons for objecting to NBS for X-ALD in general.
- Question 14: Respondents’ opinions on second-tier genetic test for X-ALD.
- Question 15-20: Demographic information.

The questionnaire was administered using Qualtrics, which was available in both Chinese and English versions. An information sheet and a three-minute short video (Supplementary File S2) were also included to provide details on clinical symptoms, diagnosis, treatment, and the potential for NBS for X-ALD. In order to aid the investigation of the public view of screening for adult-onset X-ALD, it was described that the application of NBS can detect adult-onset X-ALD. Participants were expected to gain a basic understanding of X-ALD through either the information sheet or the short video, both of which contained the same information and had been reviewed by neurologists and pathologists.

Support or opposition for NBS for X-ALD was assessed using 5-point Likert scales. Descriptive statistics were performed on Microsoft Excel (Microsoft Corporation, Redmond, WA, USA) to determine absolute frequencies and percentages for qualitative variables.

## 3. Results

A total of 259 valid responses were received. The results of all the collected responses for the 20 questions were summarized in Table A1 (Appendix A).

### 3.1. Demographics

Among the 259 collected responses, there were 94 males (36.3%) and 165 females (63.7%) (Question 15). A total of 42.5% of participants were aged between 18 and 30 years (Question 16), 100% were Chinese (Question 17), 67.2% had no religious affiliation (Question 18), and 71.4% had completed tertiary education (Question 19). Among the respondents, 196 were from the general public (75.7%) and 63 were healthcare professionals (24.3%) (Question 20). None of them were X-ALD patients, but one of the respondents was the healthcare provider for X-ALD patients. A total of 24% of the respondents (Question 3) had some level of knowledge about X-ALD before participating in this survey. However, more than half of the respondents (61.8%) were not aware of the conventional treatment (HSCT) for X-ALD (Question 4), and nearly three-quarters of the respondents (73.0%) were not aware of the new gene therapy for X-ALD (Question 5).

### 3.2. Views of NBS for X-ALD

Support for NBS:

- Support for NBS in males (Question 6): 99.2% (*n* = 257), including 75.7% (*n* = 194) of the general public and 24.3% (*n* = 63) of healthcare professionals.
- Reasons for support (Question 7): Regular monitoring and timely treatment (97.3%, *n* = 252); affordability of HSCT and hormone replacement therapy under public healthcare subsidies (72.6%, *n* = 188).
- Support for NBS in females (Questions 8 and 9): 98.5% (*n* = 255), including 75.7% (*n* = 193) of the general public and 24.3% (*n* = 62) of healthcare professionals. Given that females had late symptom onset, still, a total of 89.9% (*n* = 233) supported screening.
- Reasons for support (Question 9): Improved family support (98.1%, *n* = 254); informed reproductive decision-making (97.3%, *n* = 252); availability of symptom relief treatment (96.9%, *n* = 251); opportunity for extended family screening (94.2%, *n* = 244); and perceived unfairness of male-only screening (64.9%, *n* = 168).
- Parental decision-making (Question 11): 89.2% (*n* = 231) supported parental choice regardless of disease onset.

Non-support for NBS:

- Non-support for NBS in females: 1.5% (*n* = 4) (Question 8) of respondents did not support NBS for female newborns.

Reasons for non-support in females: Among the four respondents who did not support screening female newborns, two of them were also against screening male newborns (0.8%, *n* = 2). They strongly expressed concerns towards potentially affected insurance premiums (Question 13) as well as increased psychological stress due to uncertainty in the onset of symptoms.

Other specific reasons for supporting or not supporting NBS for X-ALD are detailed in Table A1.

Regarding the limitations of NBS for X-ALD, 66.0% (*n* = 171) of respondents believe that regular tests and monitoring would slightly to fairly impair, and 21.6% (*n* = 56) believe that it would significantly impair, the quality of life of the child with a positive screening result but unconfirmed diagnosis and unknown disease onset (Question 12). In addition, 18.9% (n = 49) of respondents agreed that parents and children would have excess psychological stress if the NBS second-tier genetic test involves variants of uncertain significance (VUS) results (Question 14).

## 4. Discussion

This cross-sectional survey is the first in Hong Kong to examine the attitudes among both the general public and healthcare professionals towards NBS for X-ALD, encompassing males with infantile- to childhood-onset disease and females with late adult-onset manifestations. Understanding the public perspectives will help to understand the support, challenges, and ethical implications that may arise if X-ALD is included in NBS. Although NBS can identify pre-symptomatic X-ALD individuals, the timing and severity of clinical onset remain difficult to predict due to the lack of genotype–phenotype correlation and disease severity biomarkers [12]. In light of these challenges, different regions have adopted diverse implementation strategies to optimize clinical outcomes (Table 1).

New York was the first state in the USA to include X-ALD in its NBS panel. The screening involves the measurement of C26:0−LPC for the first two tiers by FIA-MS/MS and then LC-MS/MS and *ABCD1* gene sequencing as the third tier. Up to March 2020, there were 16 states in the USA that started NBS for X-ALD using a similar approach to the practice in New York [10]. In stark contrast, the implementation of NBS for X-ALD in the Netherlands showed considerable differences. Advised by the Dutch Health Council, “Screening for X-ALD is useful only in male newborns, as symptoms in women usually develop later and are untreatable… The possibility to screen only male newborns without loss of efficiency should be studied [20].” Its advice was based on the World Health Organization Wilson and Jungner criteria, which dictated that NBS should be done when it brings direct health benefits [21]. With regard to the recommendation by the Dutch Health Council, the X-counter algorithm was developed and integrated as a second tier in the screening for ALD in the Netherlands study [9]. We summarize the Wilson and Jungner criteria in the context of NBS for X-ALD in Table 2.

Almost all respondents expressed a positive view of NBS for X-ALD. A total of 99.2% (*n* = 257) of respondents agreed to have their male newborns screened if NBS for X-ALD was available in Hong Kong (Question 6). Over 97% (*n* = 252) of our respondents agree that NBS for X-ALD is important for facilitating early monitoring and treatment (Question 7). A Dutch survey study of 66 respondents with X-ALD similarly reported that 80% supported NBS for both sexes, even if disease onset for females could be very late or unknown [11]. Local cross-sectional surveys have similarly reported strong support for NBS for spinal muscular atrophy (94.6%) and an NBS program for inborn errors of metabolism (83.5%) [22,23]. These findings indicate broad acceptance of NBS in Hong Kong and a shared recognition of the value of early intervention for neonatal disorders. Mahmood et al. showed that early diagnosis and timely HSCT in boys with early-stage cerebral ALD increased the 5-year survival significantly from 54% to 95% [24]. Apart from reducing mortality, early diagnosis and subsequent treatment with HSCT can also improve neurological outcomes [25]. It is estimated that NBS for X-ALD can facilitate the prevention of 37 cases (range: 17–64) of serious disability or death due to X-ALD each year for a projected annual US population of 4 million newborns back in 2015 [6]. As more jurisdictions worldwide implement NBS for X-ALD and early HSCT, the actual number of deaths and severe disability cases prevented each year is likely higher and evolving but has not yet been quantified in a consolidated global analysis.

Despite the general positive attitude observed from the responses, our respondents also expressed three major concerns revolving around NBS for X-ALD, as reflected in Question 13: the increase in psychological stress due to uncertainty of timing and onset of symptoms, the influence on insurance premiums and coverage, and the accessibility to novel gene therapy. Addressing these concerns will be conducive to future implementation of NBS programmes as well as the expansion of screening panels.

Firstly, the general public is concerned about the psychological stress brought by screening. A total of 16.2% of respondents agreed that the screening results might lead to disruption in one’s normal life (Question 13). To further elaborate, guilt from the mother (17.4%, *n* = 45) and uncertainty in disease severity and onset (21.6%, *n* = 56) are the leading causes of emotional distress. Since X-linked diseases are usually inherited from the mother, a positive screening result may give rise to a sense of guilt in the mothers. Up to 17.4% (*n* = 45) of respondents worried that the sense of guilt may escalate to bring about irreversible impacts on the parent-child relationship. Their social experience is shaped by the stigmatisation they are subject to, and discussions of this topic are often highly sensitive, leading to instabilities of interactions [26]. Apart from mothers’ biological responsibility for transmitting the disease, the responsibility to take care of their affected children further adds to their burden, and hence it is not uncommon for X-linked mothers to experience remorse related to their child’s condition, as observed in other studies [27,28]. A total of 21.6% (*n* = 56) of respondents believed that regular clinical tests and monitoring would very much impair patients’ quality of life, given that they have an unconfirmed diagnosis and unknown onset of symptoms. There is an unmet clinical need for reliable biomarkers to quantify disease severity and predict disease progression [12]. The lack of a reliable biomarker for X-ALD further intensifies the anxiety and stress due to the ambiguity of the test results that either indicate a biochemical anomaly or a potentially fatal condition [29], and the prolonged diagnostic uncertainty experienced by these patients-in-waiting is a mutual emotional distress shared by many families and healthcare providers with concerns of genetic disorders [30].

Secondly, regarding insurance, 47.9% (*n* = 124) and 8.1% (*n* = 21) of respondents agreed and strongly agreed, respectively, that the NBS results might increase their insurance premium and/or restrict coverage. In the USA, a survey conducted by the Department of Health and Human Services revealed that 25.9% of patients with genetic disorders or their family members had been refused health insurance on the basis of their genetic information [31]. In Hong Kong, one of the relevant measures is the Disability Discrimination Ordinance and the Personal Data (Privacy) Ordinance, which provides protection to a certain degree against the misuse of personal data, but similar to other Asian counterparts, the jurisdictions have not yet succeeded in specifying the lawful use of genetic information [32]. In 2020, Best Practice on the Use of Genetic Test Results was issued by the Hong Kong Federation of Insurers to prohibit insurers from asking for genetic test results for underwriting purposes (https://www.hkfi.org.hk, accessed on 1 March 2026). Nonetheless, given the unwavering public concern about genetic discrimination in the context of insurance, along with the thriving advancements made in genomic medicine, more specific regulatory measures should be set up to prevent genetic discrimination in health insurance as well as to expand coverage for follow-up care in order to pave the way for the successful implementation of NBS programmes [33].

Thirdly, a total of 33.6% (*n* = 87) and 31.3% (*n* = 81) of respondents voted agree and strongly agree, respectively, that accessibility of gene therapy is one of the deterring factors to NBS for X-ALD (Question 13). Indeed, gene therapy remains exceptionally expensive, and it may cost up to three million US dollars for the one-time medication, but it is not the only treatment option that is available [34]. In fact, most participants reported limited knowledge of available treatments for X-ALD. Over half of respondents (61.8%) were unaware of HSCT as a conventional treatment (Question 4), and nearly three-quarters (73.0%) were not aware of the newer gene therapy option (Question 5). Conventional allogeneic HSCT is limited by the availability of a human leukocyte antigen-matched donor and the risks associated with alternate donors, including severe graft-versus-host disease, prolonged immune deficiency, and potential neurological decline [35,36]. However, the alternative gene therapy in the form of autologous hematopoietic stem cell therapy with elivaldogene tavalentivec (Lenti-D) also has its limitations [37]. There are reported cases of hematological cancers developing post-treatment, especially myelodysplastic syndrome [38]. Although the financial burden is a major concern in the eyes of the general public, medical indications and risks also need to be addressed before choosing the best treatment option for the affected child. Thus, further education on the disease and treatment options is necessary to help promote screening for X-ALD.

Screening for X-ALD for female newborns was well received by our respondents, with overwhelming support of 98.5% (*n* = 255). A total of 94.2% (*n* = 244) of respondents believed that being able to identify other family members with X-ALD was a reason for supporting NBS for females (Question 9), while only 7.7% (*n* = 20) considered it unjust to screen females merely for the benefits of other family members (Question 10). A majority (97.3%, *n* = 252) of the respondents also agreed or strongly agreed that being able to help parents make informed decisions about future pregnancy was a reason for supporting NBS for females (Question 9). Overall, this suggests that in our locality, respondents may prioritize perceived immediate or family-level benefits (e.g., reproductive planning, access to care) and parental responsibility over abstract concerns about future autonomy. Given that all participants were of Chinese background, this pattern may reflect an Asian cultural emphasis on collectivism and family interests, in contrast to more individualistic value frameworks [39].

For the past two decades, there has been an ongoing debate weighing the benefits and harms of testing for adult-onset genetic conditions that are not medically actionable [40]. Genetic testing is a lifelong commitment that warrants careful evaluation, as the result will directly impact children’s “right to an open future”. On this account, that right requires that the life paths of children should not be constrained by decisions made before they are capable of giving informed consent [41]. In our survey, 32.8% (*n* = 85) of respondents endorsed preserving this right as a reason to object to NBS, and 14.6% (*n* = 38) of respondents agreed that the late age of onset and mild presentation should count against NBS for females. In contrast, a total of 38.6% (*n* = 100) still supported screening despite the late onset and mild symptoms of the disease in females, and 28.6% (*n* = 74) stayed neutral on this concern (Question 10), suggesting that many respondents do not regard the “right to an open future” argument as decisive in this context. At the same time, our survey shows that the vast majority of respondents (89.2%, *n* = 231) indicated that, hypothetically, they would want their parents to decide about X-ALD screening if they themselves were newborns, reflecting strong trust in parental decision-making and recognition of parents’ responsibility to safeguard their children’s interests (Question 11). This apparent tension between the “right to an open future” and “parental decision-making rights” deserves further exploration. In a joint policy statement, the American College of Medical Genetics and Genomics and the American Academy of Pediatrics advise against predictive testing of children for adult-onset genetic conditions, primarily to preserve the child’s “right to an open future,” while allowing limited exceptions where, after careful counseling, testing is pursued to address severe parental anxiety or anticipated psychosocial benefits. [42]. Ross’s model of “constrained parental autonomy” acknowledges parents’ primary responsibility for making decisions on behalf of their children but holds that this autonomy should be bounded by a requirement to protect the best interests of the child [43]. In the context of screening for female X-ALD, potential familial benefits, including extended family screening and future family planning, need to be balanced against harms, including the burden of knowledge without an available cure at hand and financial issues, such as eligibility for life insurance coverage. This reinforces the importance of providing sufficient information and time for parents to explore relevant ethical considerations before giving consent for NBS, especially when parents are obliged to make clinical decisions with respect to their children’s interests. In addition, as healthcare professionals, we have a fundamental responsibility to safeguard the rights and well-being of children, even when doing so may conflict with the wishes of their parents.

Several limitations of this survey should be acknowledged. First, the distribution of participants was uneven, with 75.7% drawn from the general public compared to 24.3% from healthcare professionals, and a large proportion of respondents were Chinese women without a specific religious affiliation, which may introduce bias due to the lack of demographic diversity. Despite this, the study illustrated that most people, regardless of their occupational background, would support NBS for X-ALD. As the first survey on NBS for X-ALD in Hong Kong, these findings could provide valuable insight for future studies and policy discussions. Second, our study may contain potential selection bias in the recruitment approach. As participation was based on voluntary response and influenced by the researchers’ networks, the sample may disproportionately reflect the views of individuals within similar social or professional circles. Third, the sample size was comparatively small and may limit the generalizability of the findings, underscoring the need for larger studies to validate and extend these results. Finally, the online questionnaire system does not have technical safeguards against multiple submissions from the same individual; repeat responses cannot be completely excluded. Nonetheless, the study findings serve as a strong foundation for the clinical implementation of NBS for X-ALD. 

**Figure 1:**
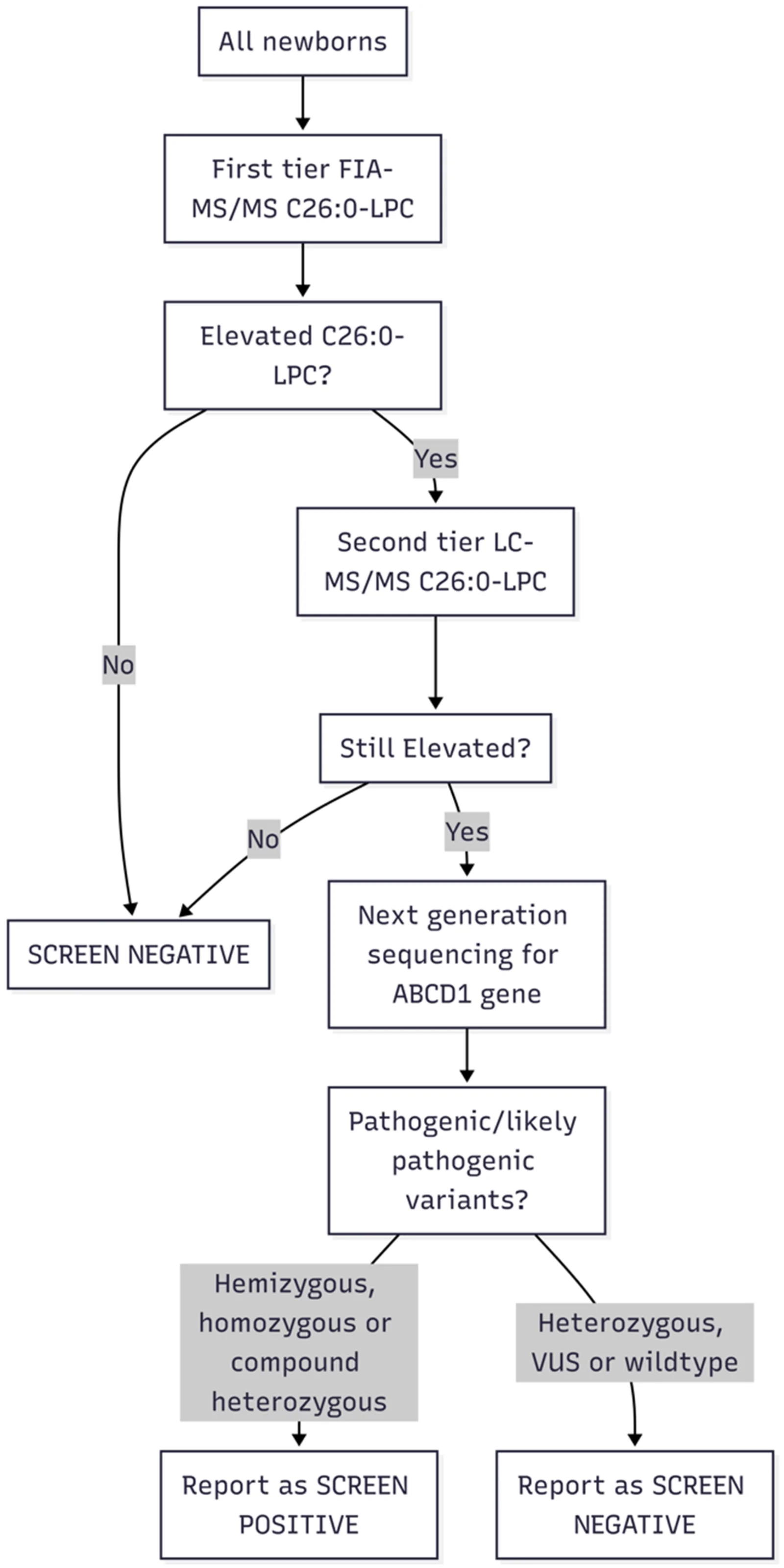
Proposed three-tier algorithm for NBS X-ALD.

To optimize the benefits of NBS for X-ALD while minimizing ethical concerns and unnecessary anxiety, we propose a targeted three-tier screening algorithm that focuses on identifying individuals at highest risk of clinically significant disease (Figure 1). All newborns first undergo FIA-MS/MS screening for elevated C26:0-LPC, followed by second-tier LC-MS/MS. Samples with persistently elevated C26:0-LPC then proceed to next-generation sequencing of the *ABCD1* gene (with optional analysis of peroxisomal biogenesis disorder genes). Only cases with likely pathogenic or pathogenic (LP/P) variants in *ABCD1* that are hemizygous, homozygous, or compound heterozygous according to the *ABCD1* Variant Registry (https://adrenoleukodystrophy.info/mutations-and-variants-in-abcd1, accessed on 1 March 2026) are reported as screen-positive. Heterozygous variants and VUS are reported as negative. This can also address newborns with Turner syndrome and Klinefelter syndrome. This approach enables early detection of high-risk male infants and rare severely affected females while avoiding routine identification of female heterozygotes with late-onset, milder phenotypes, thereby addressing key ethical, psychological, and insurance-related concerns.

## 5. Conclusions

In conclusion, our study indicates that a majority of people support the implementation of NBS for X-ALD in Hong Kong. Most participants expressed a positive attitude, believing that NBS could facilitate early diagnosis and treatment, which would contribute to better family support and improvement in the lives of X-ALD patients. There was also an overwhelming majority of 98.5% (*n* = 255) in support of female X-ALD screening. Nevertheless, several important concerns were raised: (1) psychological stress and anxiety arising from uncertain disease onset and frequent monitoring; (2) potential genetic discrimination and adverse effects on insurance premiums and coverage; (3) affordability and accessibility of ultra-expensive treatments, such as gene therapy; and (4) ethical issues regarding children’s “right to an open future”, particularly for late-onset conditions in females. These concerns highlight the tension between preserving children’s autonomy and parental decision-making rights, which deserves further exploration. To address these concerns and respect each family’s autonomy, we propose an opt-in system where parents are offered clear, balanced information and can then decide whether to include this condition in their baby’s screening. Interventions, including reinforcement in public education and government policies to prevent genetic discrimination, as well as measures to regulate insurance premium coverage, could be effective in easing the doubts. Our findings support the consideration of incorporating X-ALD into Hong Kong’s NBS program. Given the high level of public acceptance and the potential for early detection to enable timely clinical management, such an expansion may improve health outcomes for affected individuals.

## Figures and Tables

**Table 1:** NBS algorithm for X-ALD in different regions.

Regions	Screening Algorithm (C26:0-LPC Cut-Off Value (μmol/L))				Age at DBS Collection	Sex-Specific or Universal	Remarks
	Tier 1	Tier 2	Tier 3	Tier 4			
New York [10,13]	FIA-MS/MS	HPLC-MS/MS	*ABCD1* sequencing		24-36 h [14]	Universal	Recall for VLCFA and plasmalogen
Illinois [15]	HPLC-MS/MS (≥0.28)				After 24 h [16]	Universal	Recall for second DBS, plasma VLCFA, or *ABCD1* sequencing
Pennsylvania [1]	FIA-MS/MS (>0.36)	LC-MS/MS (>0.15)			24−72 h	Universal	Recall for *ABCD1* sequencing
Nebraska [17]	FIA-MS/MS (>0.36)	HPLC-MS/MS (>0.15)			24−48 h	Universal	Recall for *ABCD1* sequencing
North Carolina [18]	HPLC-MS/MS (>0.08)				24−48 h	Universal	Recall for second DBS or *ABCD1* sequencing
Italy [8]	FIA-MS/MS (≥0.5)	UHPLC-MS/MS (>0.1)			48−72 h	Universal	Recall for *ABCD1* sequencing
Taiwan [5]	FIA-MS/MS (>0.3)	HPLC-MS/MS (>0.3)	*ABCD1* sequencing		48−72 h	Universal	
Netherlands [9]	FIA-MS/MS (≥0.32)	“X-counter”	HPLC-MS/MS (≥0.15)	*ABCD1* sequencing	3–7 days old	Only one X-newborn is screened	
Japan [19]	HPLC-MS/MS				4−6 days	Only male newborns are screened	Recall for *ABCD1* sequencing and plasma VLCFA

Abbreviations: *ABCD1*, ATP-binding cassette subfamily D member 1; FIA-MS/MS, flow injection analysis with tandem mass spectrometry; HPLC-MS/MS, high-performance liquid chromatography with tandem mass spectrometry; UHPLC-MS/MS, ultra-high-performance liquid chromatography–tandem mass spectrometry; VLCFA, very-long-chain fatty acid; “X-counter”: an algorithm that determines the number of X chromosomes without assessing the presence of a Y chromosome.

**Table 2:** Wilson and Jungner screening criteria in the context of X-ALD screening.

Criteria	NBS for Males	NBS for Females
1. The condition sought should be an important health problem.	- Incidence rate of X-ALD is 1:21,000 in males. It leads to early onset and potentially fatal consequences without prompt treatment	- Incidence rate of X-ALD carriers is 1:28,000 in females. It has a slower progressive clinical manifestation and a late adulthood onset
2. There should be an accepted treatment for patients with recognized disease.	- HSCT for early-stage neurological involvement - Gene therapy for CALD - Cortisol therapy for adrenal insufficiency	- No disease-modifying therapy for AMN - Symptomatic treatment available
3. Facilities for diagnosis and treatment should be available.	- Diagnostic tests and treatment are routinely available in hospitals	- Diagnostic tests are routinely available in hospitals - Treatment limited to supportive care for AMN
4. There should be a recognizable latent or early symptomatic stage.	- X-ALD is asymptomatic at birth but detectable via VLCFA testing - Early symptomatic stage could be picked up by regular interval MRI	- Late adulthood onset or asymptomatic for life - Long latent period reduces urgency for screening
5. There should be a suitable test or examination.	- X-ALD NBS can be done by quantitative analysis of C26:0−LPC in dried blood spots	
6. The test should be acceptable to the population.	- The sample collection of a dried blood spot poses minimal risks, if any, to newborns	
7. The natural history of the condition, including development from latent to declared disease, should be adequately understood.	- The onset of disease and clinical manifestations are well-documented - Aligns with early intervention needs	- 80% of females develop AMN by the age of 60 years, with variable severity - Lack of reliable predictors of AMN severity complicates counselling
8. There should be an agreed policy on whom to treat as patients.	- There are clear guidelines on the surveillance and monitoring of male X-ALD patients	- There is no consensus on carrier management
9. The cost of case finding (including diagnosis and treatment of patients diagnosed) should be economically balanced in relation to possible expenditure on medical care as a whole.	- Low screening costs, and it leverages existing facilities for screening - Early intervention reduces long-term costs of treatment	- Same screening costs as males, but benefits are unclear - Questionable economic balance due to minimal treatment impact
10. Case finding should be a continuing process and not a “once and for all” project.	- Already integrated into ongoing NBS programmes in many places - Continuous follow-up with MRI and adrenal tests	- Sustainable via NBS - May aid family planning, but limited direct health impact

CALD: cerebral adrenoleukodystrophy.

## Data Availability

The original contributions presented in this study are included in the article/Supplementary Materials. Further inquiries can be directed to the corresponding author(s).

## Supplementary Materials

The following supporting information can be downloaded at: https://www.mdpi.com/article/10.3390/ijns12030063/s1, Supplementary File S1: X-ALD online questionnaire and consent form; Supplementary File S2: information sheet and a three-minute short video.

## Abbreviations

The following abbreviations are used in this manuscript:

ABCD1	ATP-binding cassette, subfamily D, member 1
AMN	Adrenomyeloneuropathy
CALD	Cerebral adrenoleukodystrophy
FIA-MS/MS	Flow injection analysis with tandem mass spectrometry
HPLC-MSMS	High-performance liquid chromatography with tandem mass spectrometry
HSCT	Hematopoietic stem cell transplantation
LC-MS/MS	Liquid chromatography with tandem mass spectrometry
MRI	Magnetic resonance imaging
NBS	Newborn screening
VLCFA	Very-long-chain fatty acid
VUS	Variants of uncertain significance
X-ALD	X-linked adrenoleukodystrophy

## Appendix A

**Table A1:** Summary of responses to the questionnaire (*n* = 259).

Q1. Are You a X-ALD Patient?	*n* (%)				
Yes	0 (0)				
No	259 (100)				
Q2. Do you have any relationship with X-ALD patients?	*n* (%)				
Family members or relatives	0 (0)				
Friends	0 (0)				
Legal guardian	0 (0)				
Patient and healthcare professional	1 (0.4)				
No relationship	258 (99.6)				
Others	0 (0)				
Q3. Did you know about X-ALD before this survey?	*n* (%)				
Known a lot	3 (1.2)				
Known a little	59 (22.8)				
Known nothing	197 (76.1)				
Q4. Did you know about the conventional treatment (HSCT) for X-ALD before this survey?	*n* (%)				
Known a lot	3 (1.2)				
Known a little	96 (37.1)				
Known nothing	160 (61.8)				
Q5. Did you know about the new treatment (gene therapy) for X-ALD before this survey?	*n* (%)				
Known a lot	2 (0.8)				
Known a little	68 (26.3)				
Known nothing	189 (73.0)				
Q6. If NBS for X-ALD is available in Hong Kong, would you have your male newborn screened?	*n* (%)				
Yes	257 (99.2)				
No	2 (0.8)				
Q7. To what extent do you agree with the following statements supporting newborn screening (dried blood spot test) for X-ALD in male newborn?	Strongly disagree *n* (%)	Disagree *n* (%)	Neutral *n* (%)	Agree *n* (%)	Strongly agree *n* (%)
If the screening result is positive, male newborn could undergo clinical tests and monitoring regularly, and receive treatment as soon as needed.	0 (0)	0 (0)	7 (2.7)	35 (13.5)	217 (83.8)
HSCT and steroid replacement therapy are subsidized by the public healthcare system. Families in general can afford the treatment when screened positive.	4 (1.5)	10 (3.9)	57 (22.0)	48 (18.5)	140 (54.1)
Q8. If newborn screening for X-ALD is available in Hong Kong, would you have your female newborn screened?	*n* (%)				
Yes	255 (98.5)				
No	4 (1.5)				
Q9. To what extent do you agree with the following statements supporting newborn screening for X-ALD in female newborn?	Strongly disagree *n* (%)	Disagree *n* (%)	Neutral *n* (%)	Agree *n* (%)	Strongly agree *n* (%)
Female newborns should be screened even if the symptom onset of X-ALD in females is 40–60 years old.	0 (0)	0 (0)	26 (10.0)	91 (35.1)	142 (54.8)
If the screening result is positive, diagnosis can be made more quickly when symptoms occur in adulthood.	0 (0)	0 (0)	1 (0.4)	116 (44.8)	142 (54.8)
Medications/ physiotherapy for symptom-relief are available for females when symptoms appear.	0 (0)	0 (0)	8 (3.1)	104 (40.2)	147 (56.8)
It is unfair if girls are not screened for X-ALD while boys are.	0 (0)	50 (19.3)	41 (15.8)	63 (24.3)	105 (40.5)
It enables the identification of other family members with X-ALD.	0 (0)	0 (0)	15 (5.8)	97 (37.5)	147 (56.8)
It would lead to better family support for children with X-ALD.	0 (0)	0 (0)	5 (1.9)	110 (42.5)	144 (55.6)
It would enable parents to make informed decisions about future pregnancy.	0 (0)	0 (0)	7 (2.7)	108 (41.7)	144 (55.6)
Q10. To what extent do you agree with the following statements objecting newborn screening for X-ALD in female newborn?	Strongly disagree *n* (%)	Disagree *n* (%)	Neutral *n* (%)	Agree *n* (%)	Strongly agree *n* (%)
Female rarely or never develop life-threatening symptoms.	35 (13.5)	123 (47.5)	63 (24.3)	36 (13.8)	2 (0.8)
It is unjust to the girl when she is being screened for the benefits of other family members i.e., identification of other family members with X-ALD.	36 (13.9)	119 (45.9)	84 (32.4)	20 (7.7)	0 (0)
There is no cure for adult-onset X-ALD.	21 (8.1)	114 (44.0)	71 (27.4)	53 (20.5)	0 (0)
Adult-onset diseases should not be included in NBS.	42 (16.2)	111 (42.9)	71 (27.4)	35 (13.5)	0 (0)
For adult-onset diseases, parents should reserve the autonomy of their offspring till they grow up to decide whether or not to screen.	34 (13.1)	66 (25.5)	74 (28.6)	80 (30.9)	5 (1.9)
Q11. If you were a newborn, to what extent do you agree with your parents to help you decide whether or not to screen X-ALD with the following disease onset?	Strongly disagree *n* (%)	Disagree *n* (%)	Neutral *n* (%)	Agree *n* (%)	Strongly agree *n* (%)
All onset periods.	0 (0)	0 (0)	28 (10.8)	108 (41.7)	123 (47.5)
All onset periods except the adult period.	0 (0)	3 (1.2)	63 (24.3)	77 (29.7)	116 (44.8)
Q12. To what extent do you think that regular clinical tests and monitoring would impair the following patients’ (and their parents’) quality of life?	Not impair *n* (%)	Slightly impair *n* (%)	Fairly impair *n* (%)	Very much impair *n* (%)	Extremely impair *n* (%)
Patient with confirmed diagnosis and symptom onset at childhood.	105 (40.5)	94 (36.3)	53 (20.5)	7 (2.7)	0 (0)
Patient with confirmed diagnosis, but late symptom onset (after reaching childhood).	46 (17.8)	120 (46.3)	79 (30.5)	12 (4.6)	2 (0.8)
Patient with unconfirmed diagnosis, and unknown onset of symptoms.	32 (12.4)	84 (32.4)	87 (33.6)	56 (21.6)	0 (0)
Q13. To what extent do you agree with the following statements objecting to newborn screening (dried blood spot test) for X-ALD?	Strongly disagree *n* (%)	Disagree *n* (%)	Neutral *n* (%)	Agree *n* (%)	Strongly agree *n* (%)
This screening is not necessary.	84 (32.4)	116 (44.8)	45 (17.4)	8 (3.1)	6 (2.3)
I would rather undergo tests after symptoms occur.	61 (23.6)	112 (43.2)	66 (25.5)	20 (7.7)	0 (0)
This screening makes me feel anxious.	71 (27.4)	113 (43.6)	41 (15.8)	34 (13.1)	0 (0)
Screening results pose a potential for discrimination.	53 (20.5)	117 (45.2)	60 (23.2)	29 (11.2)	0 (0)
Screening results might disrupt one’s normal life.	43 (16.6)	90 (34.7)	84 (32.4)	42 (16.2)	0 (0)
Screening results might make my life planning difficult.	58 (22.4)	111 (42.9)	61 (23.6)	29 (11.2)	0 (0)
Screening results might increase my insurance premium and/or narrow my insurance coverage.	25 (9.7)	19 (7.3)	70 (27.0)	124 (47.9)	21 (8.1)
X-linked diseases are usually inherited from the mother. Positive results may make the mother feel guilty, and/or affect the parents relationship.	40 (15.4)	102 (39.4)	72 (27.8)	45 (17.4)	0 (0)
For male patients, although gene therapy is a one-time medication, it is too expensive (3 million USD). Patients may not be able to afford the treatment even if screened positive.	13 (5.0)	37 (14.3)	41 (15.8)	87 (33.6)	81 (31.3)
Q14. To what extent do you agree with the following statement objecting to newborn screening involving genetic test for X-ALD?	Strongly disagree *n* (%)	Disagree *n* (%)	Neutral *n* (%)	Agree *n* (%)	Strongly agree *n* (%)
When the initial result involves variants of uncertain significance, this would create excess psychological stress for parents and children.	35 (13.5)	105 (40.5)	70 (27.0)	49 (18.9)	0 (0)
Q15. What is your sex?	*n* (%)				
Male	94 (36.3)				
Female	165 (63.7)				
Q16. What is your age?	*n* (%)				
18–30 years	110 (42.5)				
31–40 years	78 (30.1)				
41–50 years	43 (16.6)				
51–64 years	15 (5.8)				
65 years or above	13 (5.0)				
Q.17 What is your ethnicity?	*n* (%)				
Chinese	259 (100)				
Non-Chinese	0 (0)				
Q18. What is your religious affiliation?	*n* (%)				
No religious preference	174 (67.2)				
Christian	54 (20.8)				
Catholic	14 (5.4)				
Buddhist	15 (5.8)				
Taoist	1 (0.4)				
Hindu	0 (0)				
Muslim	1 (0.4)				
Jewish	0 (0)				
Others	0 (0)				
Q19. What is your highest level of education achieved?	*n* (%)				
Primary school	0 (0)				
Secondary school	14 (5.4)				
Post-secondary (diploma/ associate degree)	35 (13.5)				
Tertiary/ Bachelor (University)	185 (71.4)				
Master’s degree or above	25 (9.7)				
Q20. What is your occupation?	*n* (%)				
Healthcare professionals	63 (24.3)				
Others	196 (75.7)				
