# Supplementary material for: Attitudes Toward Sex-Specific Versus Universal Newborn Screening for X-Linked Adrenoleukodystrophy in Hong Kong
Source: Int J Neonatal Screen. 2026 Jul 31;12(3):63. doi: 10.3390/ijns12030063 (PMC13510819; doi:10.3390/ijns12030063)
Supplement: Supplementary file 1 [file IJNS-12-00063-s001.zip › Supplementary File S1.pdf]

Version 9, Date: 15 May, 2024

参與研究資料頁（適用於參與者）
Information Sheet to participate in a research study (For subject)

研究題目：香港公眾和醫療專業人士對新生兒腎上腺白質萎縮症篩查意見問卷調查
Research Study Title: Questionnaire on Views of Publics and Healthcare Professionals on Newborn Screening for X-Linked Adrenoleukodystrophy in Hong Kong

首席研究員：麥苗醫生 香港兒童醫院 病理學部 顧問醫生
Principal Investigator: Dr MAK, Miu Chloe, Consultant, Department of Pathology, Hong Kong Children's Hospital

我們誠邀十八歲或以上的香港居民參與是次問卷調查。在您決定您是否參與之前，請仔細閱讀下列資訊。若有任何不清楚的地方，或若您希望獲取更多資訊，請向研究人員查詢。
We would like to invite Hong Kong residents aged 18 or above to participate in this research study. Before you decide whether you should take part, please take time to read the following information carefully. If there is anything that is not clear or if you wish to obtain more information, you may contact and ask the research investigators.

研究目的 Purpose of the Study
本調查旨在探索香港公眾和醫療專業人士對新生兒腎上腺白質萎縮症篩查的意見，特別是對比普及篩查及特定性別篩查。
The goal of this study is to gather the view of the general population and healthcare professionals on newborn screening (NBS) of X-linked Adrenoleukodystrophy (XALD) in Hong Kong, specifically on Universal VS Sex-specific NBS.

過程 Procedures
您將會填寫一份線上問卷，內容有關您對於新生兒腎上腺白質萎縮症(XALD)篩查的意見，以及您的基本背景資料。該過程約需時5分鐘。
You are invited to complete an online questionnaire about your views on NBS of XALD in Hong Kong. Basic demographic information will also be collected. The process takes around 5 minutes to complete.

預期研究的持續時間 Expected Duration of Study
我們預期本研究將需要2個月完成。
We expect that this study will take about 2 months to complete.

潛在風險 Potential Risks
此研究帶來的風險（如有）很小。此研究不會收集敏感資訊或者任何可以識別到您身份的資料。此研究的結果不太可能引起任何潛在的敏感顧慮。
The study presents minimal risk, if any, to the respondents. Personal information regarding an identifiable subject is also minimal. Sensitive information would not be collected. The result of the study would be very unlikely to raise any potential sensitive concern.

潛在利益 Potential Benefits
這項研究不會為您帶來直接的利益。然而，透過參與本研究，您將更了解新生兒XALD篩查。 這項研究的結果有助於健康資訊資料庫的建立，並提高罕見疾病的管理及醫療質素。
This study will not provide direct benefits to you. However, by taking part in this study, you would gain a better understanding of NBS for XALD. The findings of this study will also help enhance the health information database development with potentials for quality improvement in healthcare management for uncommon disorders.

自願參與 / 中途退出 Voluntary Participation / Right to Withdraw
您的參與本研究純屬自願性質。在任何時候，您有權拒絕參與或隨時中途退出本研究，而無需提供理由。這將不會影響您現在及日後的常規醫療護理和服務。您會被給予足夠的時間去考慮是否參與本研究。
Your participation in this study is voluntary. You may refuse to participate or withdraw from the study at any time without giving any reason. It will not affect your present and future medical treatments and services. You will have enough time for considering the participation in this study.

保密 Confidentiality
所有數據都為匿名收集，並會被嚴格保密。只有本研究團隊才能檢閱本次調查收集的所有信息。所有電腦所存的檔案亦會有密碼保護。在我們的學術研究報告中，我們將不會用您的姓名或任何其他人的姓名，或者任何可以識別到您身份的資料。
The data will be collected anonymously. They will be kept strictly confidential. All information collected from this survey is only accessible to the research team. The information stored in the computer will be saved with password-protection. In case the results are published in academic journals, we would not disclose yours or anyone else’s name, or other identifying information.

資料保留 Data Retention
研究資料會加密形式儲存在醫管局網路電腦中，並在研究完成3年後銷毀。
The depersonalized data will be stored in HA intranet computer, in encrypted format, and will be deleted permanently 3 years after completion of study.

參與研究的報酬Payments if participating in the study
您不會因參與本次問卷調查而獲得任何報酬。
You will not receive any payment for participating in this questionnaire survey.

問題和疑慮 Questions and concerns
如果您對研究有任何疑問，請聯絡麥苗醫生(電話:3513 3018)。若您對作為研究參加者所享有的權利有任何疑問，您可致電3513 3193與醫院管理局中央研究倫理委員會聯絡。
If you have any questions about the research, please contact Dr MAK, Miu Chloe at 3513 3018. If you have questions related to your rights as a research participant, please contact Hospital Authority Central Institutional Review Board at 3513 3193.

1. 我已閱讀並理解以上資訊。我明白所有有關本研究的好處及風險。我有機會向研究人員提出疑問，而他/她亦已完滿地解答我的疑問。對於此研究，我已獲得足夠的資料。I have read and understand this document. I understand all the benefits and risks related to this study. I have opportunities to raise questions to the investigators and they have answered my questions to my satisfaction. I have collected sufficient information regarding this study.

☐ 是 Yes
☐ 否 No

2. 我同意參與這項研究。I agree to participate in the above study.

☐ 是 Yes
☐ 否 No

線上問卷Online Questionnaire

參與者背景 **Participant Background**

1. 您是否患有 XALD? Are you a XALD patient?

☐ 是 Yes
☐ 否 No

2. 您與 XALD 患者有關係嗎? Do you have any relationship with XALD patients?

☐ 家人和/或親戚 Family members and/or relatives
☐ 朋友 Friends
☐ 法定監護人 Legal guardian
☐ 醫護關係 Patient and healthcare professional
☐ 沒有關係 No relationship
☐ 其他 Other: ______

3. 您在本次調查之前了解 XALD 嗎? Did you know about XALD before this survey?

☐ 我已經知道很多 I have already known a lot
☐ 我只知道一點 I only knew a little
☐ 我什麼都不知道 I didn't know anything

4. 您知道 XALD 的傳統療法(骨髓移植)嗎? Did you know about the conventional treatment (HSCT) for XALD?

☐ 我已經知道很多 I have already known a lot
☐ 我只知道一點 I only knew a little
☐ 我什麼都不知道 I didn't know anything

5. 您知道 XALD 的新療法(基因療法)嗎? Did you know about the new treatment (Gene therapy) for XALD?

☐ 我已經知道很多 I have already known a lot
☐ 我只知道一點 I only knew a little
☐ 我什麼都不知道 I didn't know anything

參與者意見 **Participant Views**

資訊回顧：
- 男性XALD患者多數在約2-20歲出現嚴重的大腦/腎上腺症狀。
- 造血幹細胞移植或基因療法可根治早期的大腦症狀。
- 類固醇補充療法可控制腎上腺症狀。

Brief recap:
- Severe cerebral/adrenal symptoms appear around 2-20 years old for male XALD patients.
- Early onset cerebral symptoms could be cured by HSCT or gene therapy.
- Adrenal insufficiency could be monitored by steroid replacement therapy.

6. 如果香港實行新生兒 XALD 篩查，您會讓您的兒子接受篩查嗎? If newborn screening for XALD is available in Hong Kong, would you have your male child screened?

☐ 會。Yes.
☐ 不會。No.

7. 您有多認同以下支持男嬰進行XALD篩查的原因? To what extent do you agree with the following statements supporting newborn screening for XALD in male newborn? (1=完全不同意, 5=完全 同意 1=strongly disagree, 5=strongly agree)

陳述 Statements	看法 Opinion (1-5)
a. 如果篩查結果為陽性，可為男嬰進行定期臨床評估和監察，並在有需要時盡快治療。If the screening result is positive, male newborn could undergo clinical tests and monitoring regularly, and receive treatment as soon as needed.	
b. 骨髓移植及類固醇補充療法會由醫管局資助，若篩查結果為陽性，普遍家庭亦能負擔治療費用。HSCT and steroid replacement therapy are subsidized by the Hospital Authority. Families in general can afford the treatment when screened positive.	

資訊回顧：
- 女性XALD基因攜帶者多數在約40-60歲出現慢性的神經退化症狀（AMN）。如肌肉無力、感覺失調、排泄困難...
- AMN無法被根治，但能通過物理治療和/或藥物治療舒緩症狀。

Brief recap:
- For females with XALD, slow progressive spinal cord dysfunction symptoms (AMN) appear around 40-60 years old. E.g. muscle weakness, sensory dysfunction, difficulties in excretion…
- AMN could not be cured. Medication and/or physiotherapy are available for symptom relief.

8. 如果香港實行新生兒 XALD 篩查，您會讓您的女兒接受篩查嗎? If newborn screening for XALD is available in Hong Kong, would you have your female child screened?

☐ 會。Yes.

☐ 不會。No.

9. 您有多認同以下支持女嬰進行XALD篩查的原因? To what extent do you agree with the following statements supporting newborn screening for XALD in female newborn? (1=完全不同 5=完全同意 1=strongly disagree, 5=strongly agree)

陳述 Statements	看法 Opinion (1-5)
a. 即使女性XALD患者在40-60歲才發病，也應該為女嬰進行篩查。Female newborns should be screened even if the symptom onset of XALD in females is 40-60 years old.	
b. 如果篩查結果為陽性，當症狀在成年后出現，她能更快得到診斷。If the screening result is positive, diagnosis can be made more quickly when symptoms occur in adulthood.	
c. 當症狀出现后，女性能接受物理治療/藥物治療以舒緩症狀。Medications/physiotherapy for symptom-relief are available for females when symptoms appear.	
d. 僅篩查男嬰是對女嬰的不公平。It is unfair if girls are not screened for XALD while boys are.	
e. 這有助識別其他家庭成員是否患有 XALD。It enables the identification of other family members with XALD.	
f. 這有助家庭對患有XALD的兒女提供更好的支持。It would lead to better family support for children with XALD.	
g. 這有助家長對未來生育作出知情決定。It would enable parents to make informed decisions about future pregnancy.	

10. 您有多認同以下反對女嬰進行XALD篩查的原因? To what extent do you agree with the following statements objecting newborn screening for XALD in female newborn? (1=完全不同意, 5=完全同意 1=strongly disagree, 5=strongly agree)

陳述 Statements	看法 Opinion (1-5)
a. 女性很少/不會出現危及生命的症狀。Females rarely or never develop life-threatening symptoms.	
b. 為了其他家庭成員的利益（識別其他家庭成員是否患有 XALD）而令女嬰接受篩查是不公平的。It is unjust to the girl when she is	

being screened for the benefits of other family members i.e. identification of other family members with XALD.	
c. 成年后發病的XALD無法被根治。There is no cure for adult-onset XALD.	
d. 成年后發病的疾病不應被納入新生兒篩查。Adult-onset diseases should not be included in NBS.	
e. 對於成年發病的疾病, 父母應保留子女的自主權, 待他們長大后自行決定是否願意進行篩查。For adult onset diseases, parents should reserve the autonomy of their offspring till they grow up to decide whether or not to screen.	

11. 您有多願意在您仍是嬰兒時，讓您的父母代你決定是否接受以下發病期的XALD篩查? If you were a newborn baby, to what extent do you agree with your parents to help you decide whether or not to screen XALD with the following disease onset? (1=完全不願意, 5=完全願意 1=strongly disagree, 5=strongly agree)

陳述 Statements	看法 Opinion (1-5)
a. 所有發病期。All onset periods.	
b. 所有發病期, 除了成年期。All onset periods except adult period.	

12. 您認為定期臨床評估和監察會多大程度上損害以下患者(及其家長)的生活質素？To what extent do you think that regular clinical tests and monitoring would impair the following patients' (and their parents') quality of life? (1=不會損害, 5=嚴重損害 1= not impair, 5=severely impair)

陳述 Statements	看法 Opinion (1-5)
a. 得到確診及症狀於幼年出現的患者。Patient with confirmed diagnosis and symptom onset at childhood.	
b. 得到確診, 但症狀於成年後才出現的患者。Patient with confirmed diagnosis, but late symptom onset (after reaching adulthood).	
c. 篩查陽性但未能確診, 且未知何時會有症狀的患者。Patient with unconfirmed diagnosis, and unknown onset of symptoms.	

資訊回顧
- XALD的新生兒篩查可通過干血斑測試進行。
- 干血斑測試由嬰兒腳跟取血斑樣本, 並量度酶体指標。
- 有陽性篩查結果的人需進行血液檢測來診斷XALD。

- 血液檢測包括長鏈脂肪酸 (VLCFA)分析和ABCD1基因分析。

Brief recap:

- NBS for XALD can be done by the dried blood spot test, where blood spots are taken from the baby’s heel for enzyme quantification.
- Individuals with positive NBS results will undergo blood tests for a definitive diagnosis.
- The blood test includes very long-chain fatty acid (VLCFA) analysis and ABCD1 gene analysis.

13. 您有多認同以下反對新生兒XALD篩查(乾血斑酶體)的原因? To what extent do you agree with the following statements objecting newborn screening (dry blood spot test) for XALD? (1=完全不同意, 5=完全 意 1=strongly disagree, 5=strongly agree)

陳述 Statements	看法 Opinion (1-5)
a. 這項篩查沒有必要進行。This screening is not necessary.	
b. 我寧願在症狀出現後才去進行相關檢查。 I would rather undergo tests after symptoms occur.	
c. 這個篩查會讓我焦慮。This screening would make me anxious.	
d. 篩查結果有機會讓我受到歧視。Screening results pose a potential for discrimination.	
e. 篩查結果或會對生活造成困擾。Screening results might disrupt one's normal life.	
f. 篩查結果或會令我難以規劃人生。Screening result might make my life planning difficult.	
g. 篩查結果或會提高保險費用和/或縮窄保險範圍。Screening results might increase my insurance premium and/or narrow my insurance coverage.	
h. X染色體的疾病多數由母親遺傳，陽性篩查結果可能會令母親愧疚和/或影響父母關係。X-linked diseases are usually inherited from the mother. Positive results may make the mother feel guilty, and/or affect the parents relationship.	
i. 對於男性XALD患者，基因療法雖是一生一次的治療，但過於昂貴(約港幣2千萬元)，現在亦未受公營醫療資助，患者不能負擔治療費用。For male patients, although gene therapy is a one-time medication, it is too expensive (HKD 20 million). Patients may not be able to afford the treatment even if screened positive.	

資訊回顧

- ABCD1基因有超過1000種變異方式，包括約950種致病變異、約250種不確定意義的變異(VUS)、及約40種良性變異。
- 若檢測出 VUS，可能需花費更多時間及金錢進行檢查，以作出診斷。
- 就算檢測出致病基因，亦未必能判斷該基因的臨床表現症狀。
- 現時缺乏生物指標在症狀出現前判斷XALD的嚴重程度。

Brief recap:

- There are over 1000 variants in ABCD1 gene, including around 950 pathogenic variants, 250 variants of unknown significance (VUS), and 40 benign variants.
- When VUS are detected, extra time and costs would be spent on tests and check-ups to confirm diagnosis.
- The clinical presentation may not be determined even when pathogenic variants are detected.
- There is a lack of biomarkers to predict disease severity of XALD before symptoms occur.

14. 您有多認同以下反對新生兒XALD篩查（基因分析）的原因? To what extent do you agree with the following statements objecting newborn screening (genetic test) for XALD? (1=完全不同意, 5=完全 意 1=strongly disagree, 5=strongly agree)

陳述 Statements	看法 Opinion (1-5)
a. 若檢測出不確定意義的基因變異，進行其他檢查後發現嬰兒為健康，會白白浪費時間及金錢。When the initial result is VUS, time and costs would be wasted if the child is confirmed healthy after other tests and check-ups.	
b. 等待診斷的過程會對孩子和家長造成過多的心理壓力。The pre-confirmation processes create excess psychological stress for parents and children.	

參與者基本資料 **Basic Demographic Information**

15. 您的性別是? What is your sex?

- ☐ 男性 Male
- ☐ 女性 Female
- ☐ 其他 Other:_____

16. 您的年齡是 (歲)? What is your age (years)?

- ☐ 18-30
- ☐ 31-40
- ☐ 41-50
- ☐ 51-64
- ☐ 65歲或以上 65 years old or above

17. 您的種族是?What is your ethnicity?

☐ 華人 Chinese

☐ 其他 Other: _____

18. 您的宗教信仰是? What is your religious affiliation?

☐ 沒有任何宗教信仰 None, I have no religious preference

☐ 基督教 Christian

☐ 天主教 Catholic

☐ 佛教 Buddhist

☐ 道教 Taoist

☐ 印度教 Hindu

☐ 伊斯蘭教 Muslim

☐ 猶太教 Jewish

☐ 其他 Other: _____

19. 請問您的最高教育程度是什麼? What is your highest level of education achieved?

☐ 小學程度或以下 Primary school

☐ 中學程度 Secondary school

☐ 大專程度 (文憑/副學士學位)Post-secondary (diploma / associate degree)

☐ 大學程度 Tertiary / Bachelor (University)

☐ 碩士程度或以上 Master's degree or above

20. 您的職業是? What is your occupation?

☐ 醫護人員 Healthcare provider

☐ 其他 Other:___

21. 您是否育有子女? Do you have children?

☐ 是。Yes.

☐ 否。No.

22. 您對本研究和/或XALD新生兒篩查有沒有其他意見? (非必填) Do you have any other opinions for this study and/or NBS for XALD? (Not mandatory)

感謝您的參與。
如果您對研究有任何疑問，請聯絡麥苗醫生(電話：35133018)。若您對作為研究參加者所享有的權利有任何疑問，您可致電3513 3193與醫院管理局中央研究倫理委員會聯絡。
If you have any questions about the research, please contact Dr MAK, Miu Chloe at 35133018. If you have questions related to your rights as a research participant, please contact Hospital Authority Central Institutional Review Board at 3513 3193.
