# Supplementary material for: Attitudes Toward Sex-Specific Versus Universal Newborn Screening for X-Linked Adrenoleukodystrophy in Hong Kong
Source: Int J Neonatal Screen. 2026 Jul 31;12(3):63. doi: 10.3390/ijns12030063 (PMC13510819; doi:10.3390/ijns12030063)
Supplement: Supplementary file 1 [file IJNS-12-00063-s001.zip › Supplementary File S2.pdf]

X-LINKED ADRENOLEUKODYSTROPHY (XALD) — BACKGROUND INFORMATION

What is XALD?

XALD is a genetic disorder caused by ABCD1 gene mutation on the X chromosome.

Inheritance

As female carriers have milder symptoms with later disease onset, XALD is usually inherited from the mother.

Among the offspring of a female carrier and a normal male, boys have a 50% chance of developing XALD whilst girls have a 50% chance of becoming a carrier;

Among the offspring of a male patient, all girls are carrier.

Symptoms and Treatment

Patients with XALD are **born without symptoms**. Males and females have **different disease onset** and **severity**.

Female Carrier:

- With one more X chromosome to compensate metabolic functions, symptoms appear **later in life** and in **milder** forms.
- Symptoms of **spinal cord dysfunction (AMN)** appear around **40-60 years old**. E.g. muscle weakness, sensory dysfunction, difficulty in excretion...

Conventional Treatment:

- AMN symptoms can be relieved by **medications** and/or **physiotherapy**.

Male Patient:

- Symptoms appear **earlier in life** and in **more severe** forms.
- Up to **1/3** patient **below the age of 12** develop cerebral demyelination, which cause **potentially fatal cerebral symptoms** such as epilepsy.
- **80%** patient develop **adrenocortical insufficiency before adulthood**.
- They may also develop **AMN** similar to females.

Conventional Treatment:

- Early detected cerebral symptoms can be managed by **haematopoietic stem cell transplant (HSCT)**.
- Adrenal gland problems can be monitored by **steroid replacement therapy**.

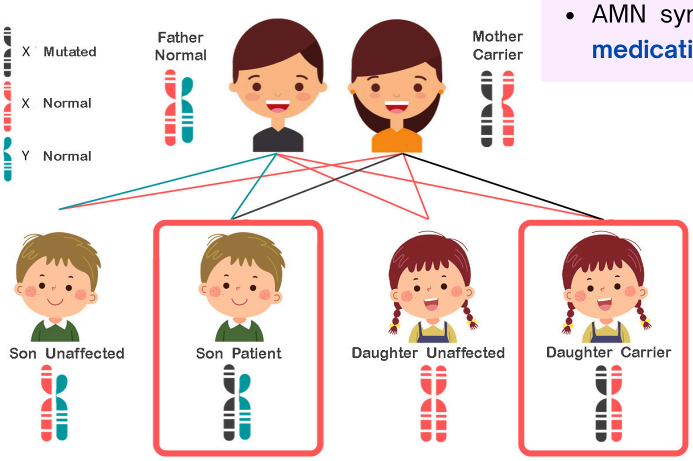

New Treatment - Gene Therapy:

- In 2022, The Food and Drug Administration granted approval of a new **gene therapy (Skysona)** to treat the **early onset neurological symptoms**.
- Its long term efficacy and adverse effect are yet to be analysed by clinical trials.
- It is not on the HA Drug Formulary, hence need to be self-purchased by the patient and/or subsidised by other charity group.
- It is a one-time medication at a price of around HKD 20 million.

Newborn Screening and Diagnosis of XALD

- NBS for XALD can be done by the **dried blood spot test**. (Blood spots taken from the baby's heel for enzyme quantification).
- Individuals with positive NBS results will undergo **blood test** for a definitive diagnosis. The blood test includes very long-chain fatty acid (VLCFA) analysis and ABCD1 gene analysis.
- NBS for X-ALD is available in many parts of the world including Dutch, New York State, Taiwan, and Italy, with Dutch (Netherland) screening exclusively males, while the others screen both males and females.
- In Hong Kong, a pilot NBS programme was launched by the CUHK-BCM Joint Centre for Medical Genetics to provide NBS for 31 inborn errors of metabolism (IEMs), with X-ALD being added to the panel in September 2021.

Benefits of Screening:

- It enables diagnosis as early as possible, and start treatment when needed. This maximises disease controllability, and improve patient's life.
- It would enable parents to make informed decisions about future pregnancy.
- Positive results may indicate cascade family screening, which help identify family members with XALD.

Limitations of Screening:

- There are over 1000 variants in ABCD1 gene，including around 950 pathogenic variants, 250 variants of unknown significance (VUS), and 40 benign variants.
- When VUS are detected, extra time and costs would be spent on tests and check-ups to confirm diagnosis.
- There is a lack of studies to define genotype-phenotype correlation of XALD. The clinical presentation may not be determined even when pathogenic variants are detected.
- There is a lack of biomarkers to predict disease severity of XALD before symptoms occur.
- The pre-confirmation process may create excess psychological stress for parents and children.

X-連鎖的腎上腺腦白質營養不良 (XALD) — 背景資料

甚麼是XALD?

XALD是由X染色體上ABCD1基因突變引起的遺傳性疾病。

XALD是怎樣遺傳的?

由於女性基因攜帶者的症狀較輕微且較遲出現，該疾病多數由女性遺傳至下一代。

若母親是基因攜帶者，父親有正常基因，他們的兒子有50%機會患上XALD，女兒有 50%機會成為基因攜帶者；

若父親是XALD患者，則女兒一定會是基因攜帶者 。

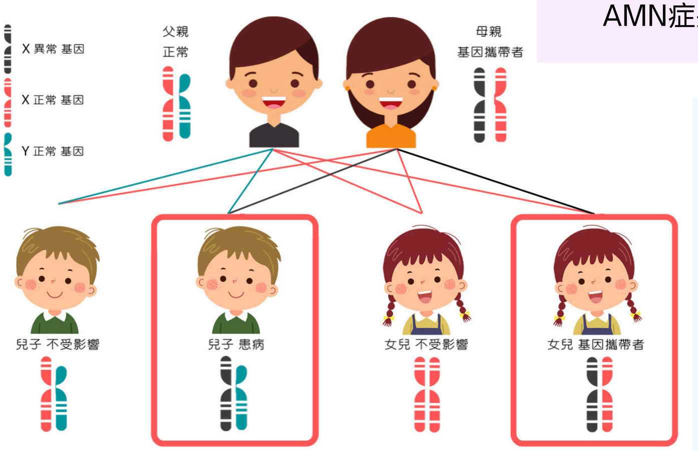

XALD有甚麼症狀？如何治療？

XALD患者在**出生時沒有症狀**。男性和女性有**不同病發期**及症狀的**嚴重程度**。

女性（基因攜帶著）:

- 擁有兩條X染色體，若其中一條攜帶突變基因，另一條正常X染色體仍能支配部分功能，因此症狀**較輕微**且**較遲出現**。
- 多數在**40-60歲**出現慢性的**神經退化**症狀（俗稱**AMN**），如肌肉無力、感覺失調、排泄困難等。

傳統療法:

- **物理治療**和/或**藥物治療**可舒緩AMN症狀。

男性（患者）的症狀:

- 只有一條X染色體，若攜帶突變基因，症狀**較嚴重**且於**早期出現**。
- 約 **1/3** 患者會在**12歲前**出現**腦白質化**的神經症狀，例如癲癇，**嚴重者會引致死亡**。
- **八成**患者會在**成年前**出現**腎上腺功能缺失**。
- 有機會在**40-60歲**出現**AMN**症狀。

傳統療法:

- **造血幹細胞移植**可治療早期發現的大腦症狀。
- **類固醇補充**可控制腎上腺症狀。

新療法 — 基因療法:

- 2022年，**基因療法Skysona**獲美國食品藥物管理局批准，用於治療**早期的大腦及神經系統症狀**。
- 它的長期治療成效及長期副作用仍有待觀察，且未被納入香港醫管局藥物名單，需由**患者自費購買/其他關愛基金資助**。
- 基因療法是一種**一次性**的藥物治療。
- 該藥物的售價約為**港幣2千萬元**。

XALD的新生兒篩查及診斷

- XALD的新生兒篩查可通過**干血斑測試**進行。(由嬰兒腳跟取血斑樣本，量度酶体指標)
- 有陽性篩查結果的人需進行**血液檢測**來診斷XALD。血液檢測包括長鏈脂肪酸（VLCFA）分析和ABCD1基因分析。
- 包括荷蘭、紐約州、臺灣和意大利在內的許多地區提供XALD的新生兒篩查。其中荷蘭僅對男性進行篩查，其他地區則對男女都進行篩查。
- .在香港，香港中文大學-貝勒醫學院聯合醫學遺傳學中心推出了新生兒篩查先行計劃，為31種先天性代謝疾病提供篩查，其中XALD於2021年9月納入篩查範圍。

篩查的好處:

- 及早診斷及治療，最大程度地控制病情，改善生活。
- 有助家長計畫未來生育。
- 陽性結果指向家族基因篩查，有助識別其他家庭成員是否患有 XALD。

篩查的限制:

- ABCD1基因有超過1000種變異方式，包括約950種致病變異、約250種不確定意義的變異 (VUS)、及約40種良性變異。
- 若檢測出 VUS , 可能需花費更多時間及金錢進行檢查，以作出診斷。
- 就算檢測出致病基因，亦未必能判斷該基因的臨床表現症狀。
- 現時缺乏生物指標以在症狀出現前判斷XALD的嚴重程度。
- 等待診斷的過程或會對孩子和家長造成過多的心理壓力。

Education video on NBS for X-ALD

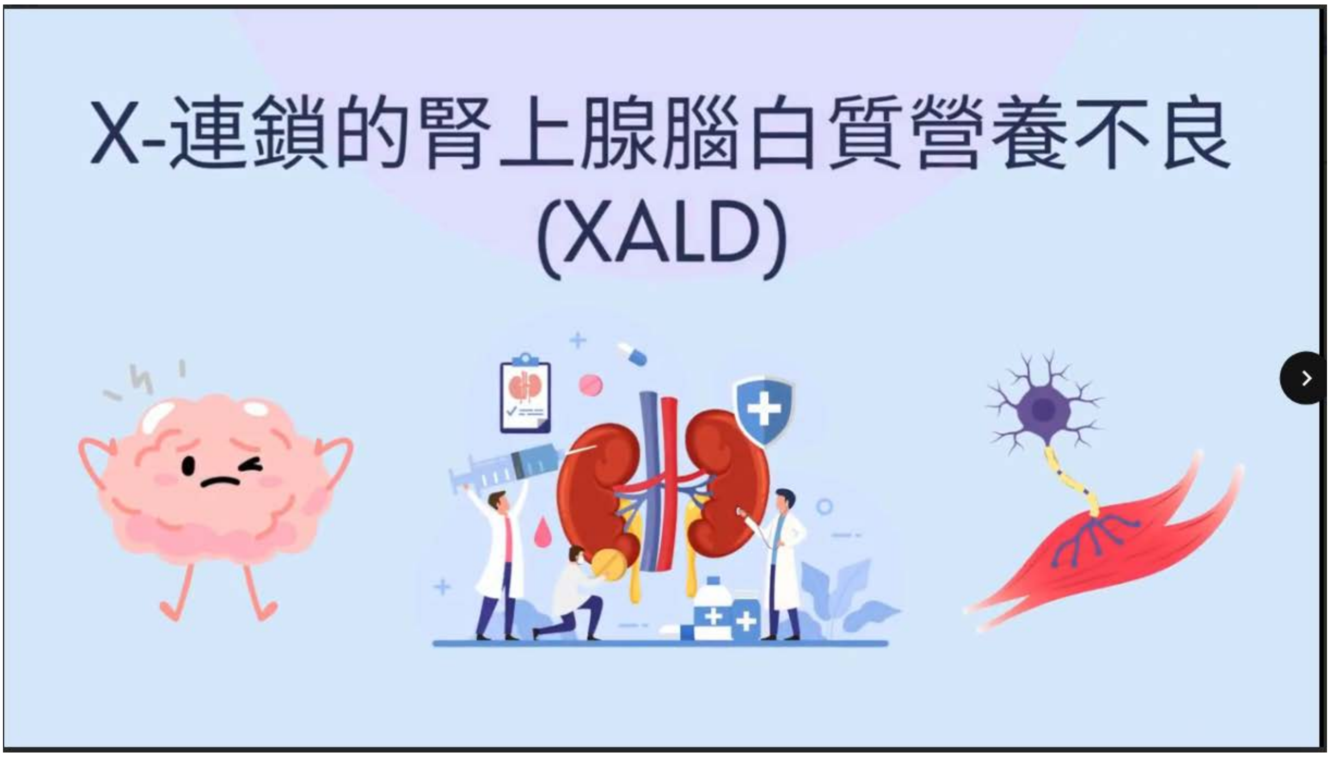

Accessed on https://drive.google.com/drive/folders/1SsSGFwVG1ITR4_ewyJTlQAqYAvUwYIhY
